# Supplementary material for: Nuclear pore protein NUP210 depletion suppresses metastasis through heterochromatin-mediated disruption of tumor cell mechanical response
Source: Nat Commun. 2021 Dec 13;12:7216. doi: 10.1038/s41467-021-27451-w (PMC8669001; doi:10.1038/s41467-021-27451-w)
Supplement: Supplementary file 1 — Supplementary Information [file 41467_2021_27451_MOESM1_ESM.pdf]

Supplementary Information

Nuclear pore protein NUP210 depletion suppresses metastasis through heterochromatin-mediated disruption of tumor cell mechanical response

Amin *et al*.

Supplementary Fig. 1

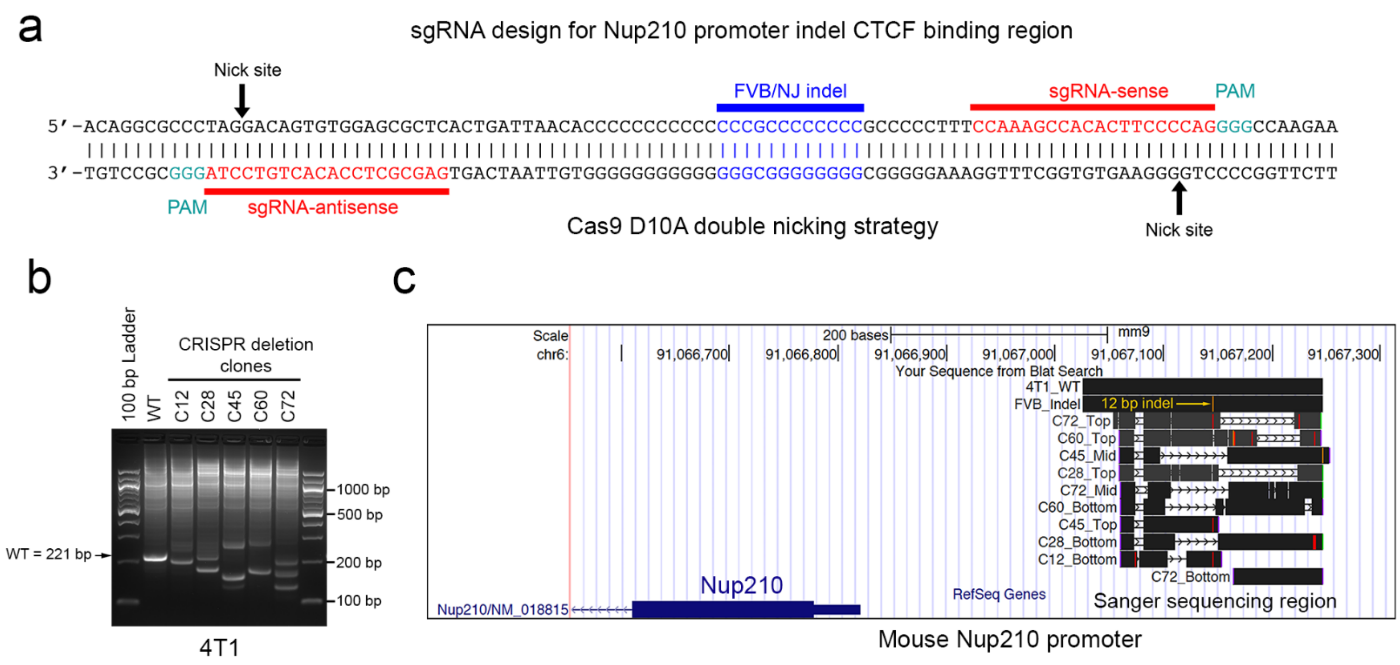

Supplementary Fig. 1: CRISPR/Cas9-mediated deletion of CTCF binding site on *Nup210* promoter.

(a) sgRNA design strategy for the deletion of putative CTCF binding site on Nup210 promoter of 4T1 (BALB/cJ-derived) cells. FVB/NJ 12 bp indel region on CTCF binding site was shown in blue.

(b) PCR amplification of 221 bp region spanning the Cas9 D10A-deleted CTCF binding site in 4T1 cell clones.

(c) UCSC genome browser view of the DNA sequences of individual band (top/mid/bottom) obtained from each clone mentioned in (b). Line with arrow indicates mutated region.

Supplementary Fig. 2

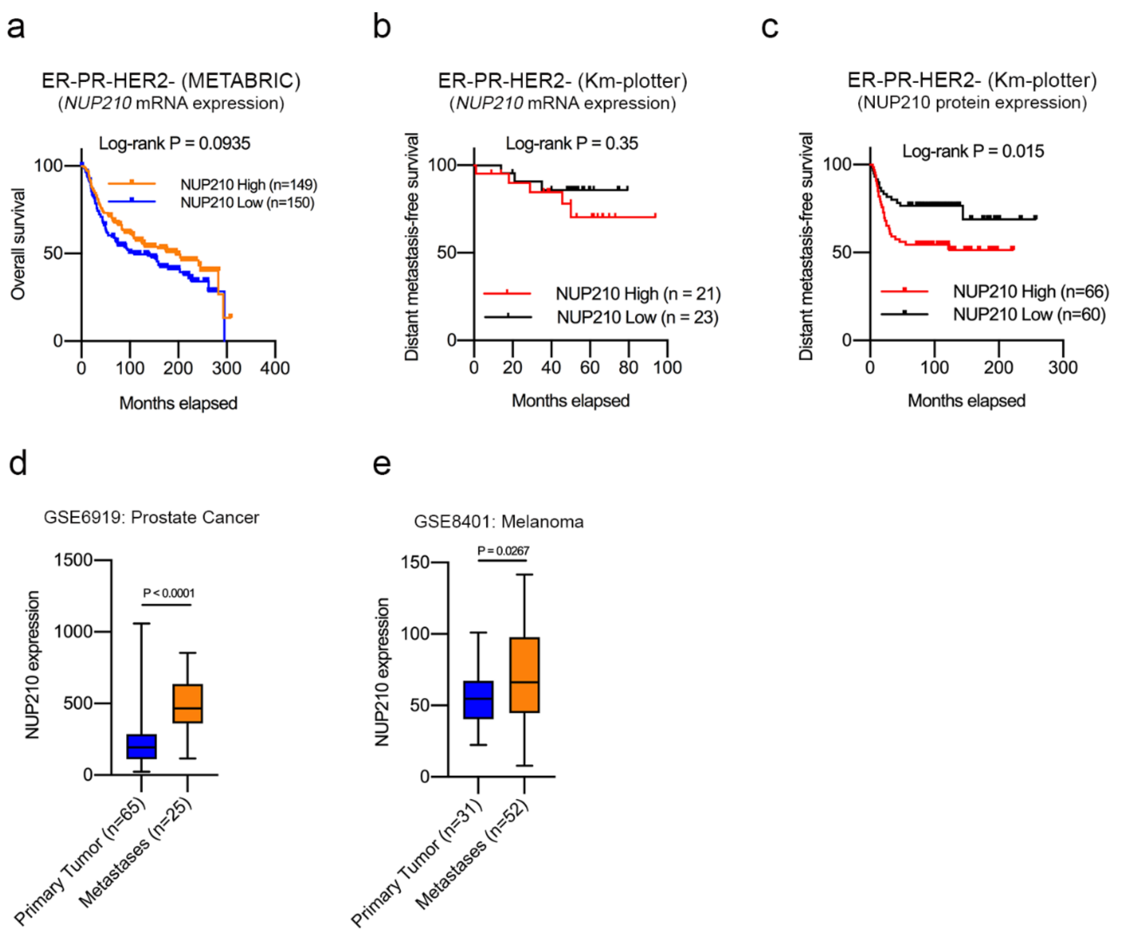

Supplementary Fig. 2: Association of NUP210 expression in triple negative (ER-/PR-/HER2-) patient and NUP210 expression in human metastases datasets.

(a) Association of NUP210 mRNA with overall survival and (b) DMFS in ER-/PR-/HER2- patients.

(c) Association of NUP210 protein level on distant metastasis-free survival of ER-/PR-/HER2- patient.

(d) Human prostate cancer dataset showing the differential expression of *NUP210* mRNA between primary tumor and metastases. Mann-Whitney U test, the box extends from 25th to 75th percentile, whiskers extend from smallest values to the largest values, horizontal line represents median.

(e) Human melanoma dataset showing the differential expression of *NUP210* mRNA between primary tumor and metastases. Mann-Whitney U test, the box extends from 25th to 75th percentile, whiskers extend from smallest values to the largest values, horizontal line represents median.

Supplementary Fig. 3

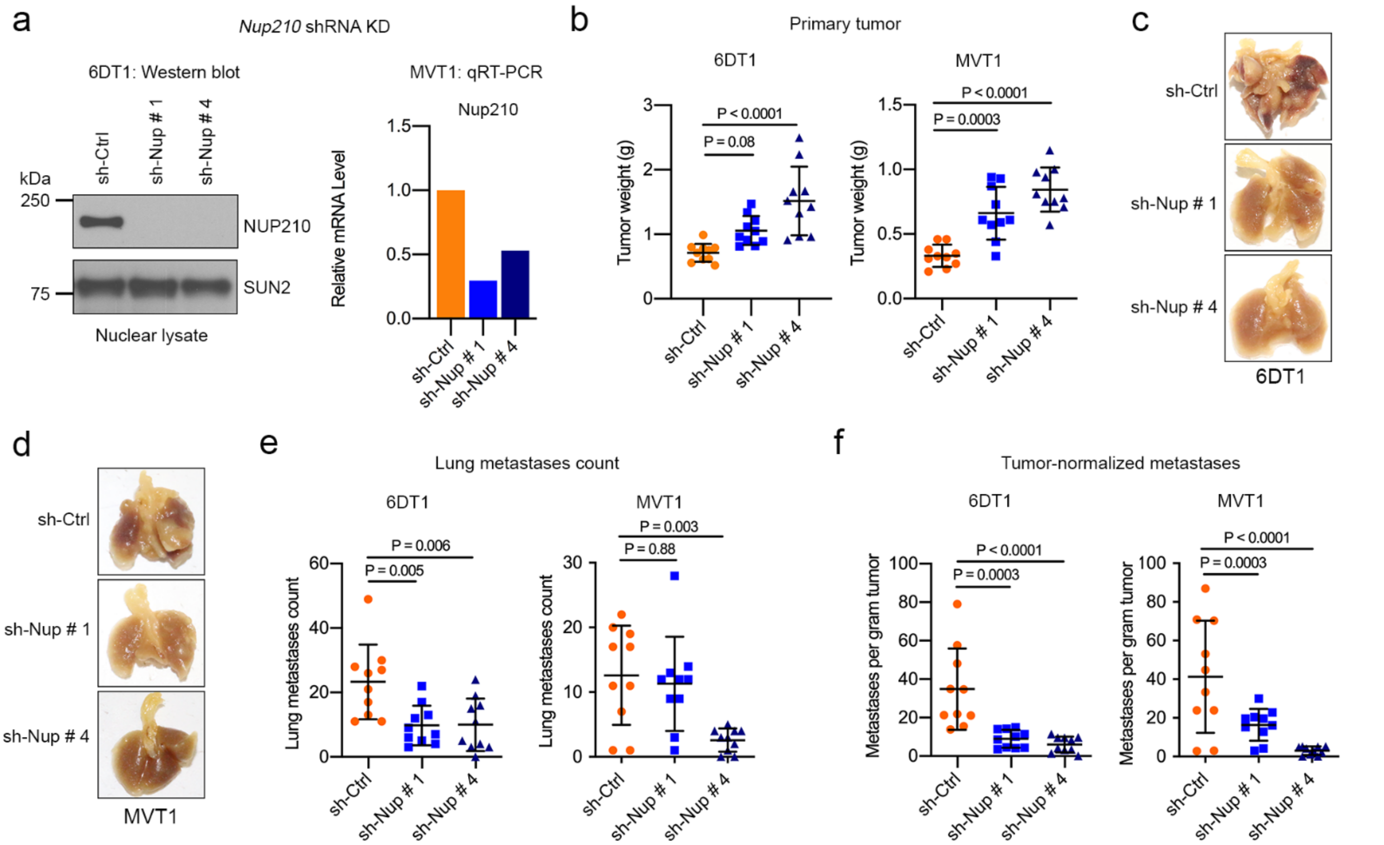

Supplementary Fig. 3: Knockdown of *Nup210* in the 6DT1 and MVT1 cell lines decreases lung metastasis.

(a) (Left) western blot NUP210 protein of *Nup210* KD 6DT1 cells and (right) qRT-PCR of *Nup210* KD level in MVT1 cells.

(b) Primary tumor weight after orthotopic transplantation of *Nup210* KD 6DT1 (left) or MVT1 (right) cells. ANOVA with Tukey's multiple comparison test, mean ± s.d. n = 10 mice per group.

(c) Representative lung images of the mice injected with *Nup210* KD 6DT1 cells.

(d) Representative lung images of the mice injected with *Nup210* KD MVT1 cells.

(e) Lung metastases count after orthotopic transplantation of *Nup210* knockdown 6DT1 (left) and MVT1 (right) cells. ANOVA with Tukey's multiple comparison test, mean ± s.d. n = 10 mice per group.

(f) Lung metastases count normalized to primary tumor weight, derived from values shown in (b) and (e). ANOVA with Tukey's multiple comparison test, mean ± s.d. n = 10 mice per group.

Supplementary Fig. 4

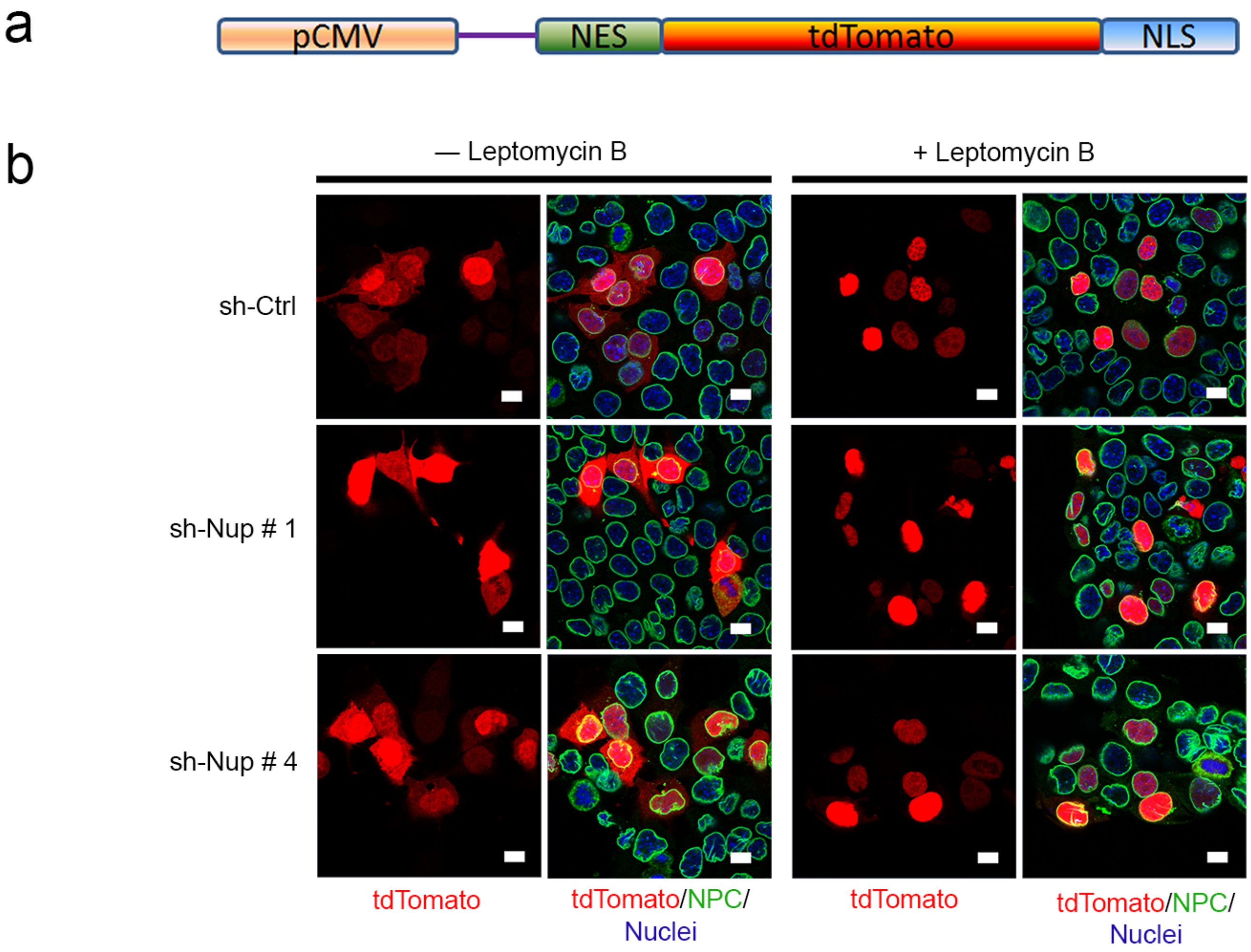

Supplementary Fig. 4: NUP210 loss does not affect general nucleocytoplasmic protein transport.

(a) Schematic of the tdTomato-expressing nucleocytoplasmic transport reporter. Reporter expression is driven by a CMV promoter and produces tdTomato fused to both a nuclear export (NES) and import (NLS) signal.

(b) Representative immunofluorescence images showing nuclear and cytoplasmic localization of the tdTomato signal in sh-Ctrl and *Nup210* KD 4T1 cells. The Nuclear Pore Complex (NPC) is shown in green. Nuclei are stained with DAPI. Leptomycin B, a nuclear export inhibitor drug, was used as positive control. Scale bar = 10 µm.

Supplementary Fig. 5

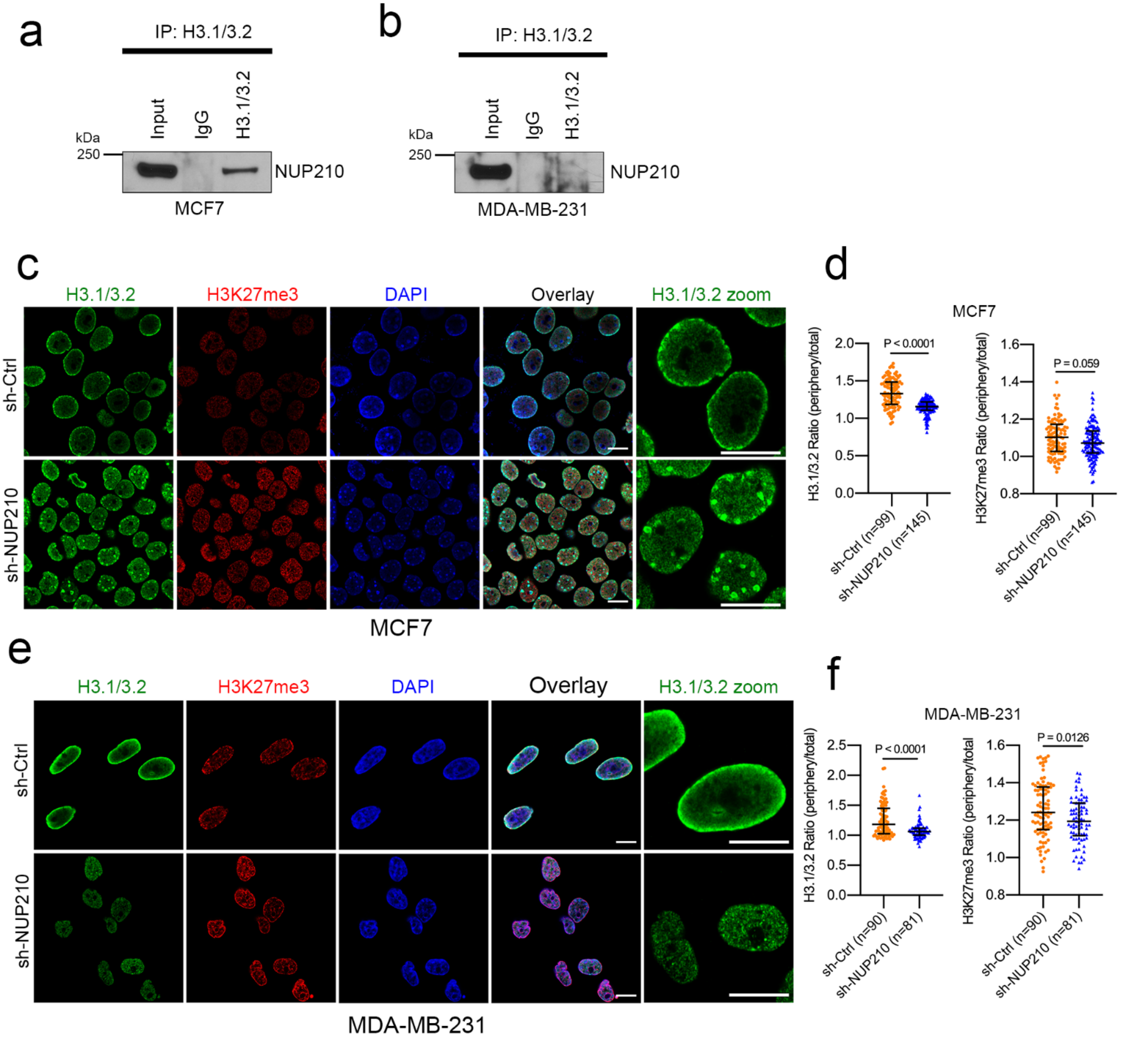

Supplementary Fig. 5: NUP210 interaction with H3.1/3.2 in human breast cancer cell lines MCF7 and MDA-MB-231.

(a) Co-IP of NUP210 and H3.1/3.2 in MCF7 cell line.

(b) Co-IP of NUP210 and H3.1/3.2 in MDA-MB-231 cell line.

(c) Immunofluorescence staining of H3.1/3.2 and H3K27me3 in NUP210 knockdown MCF7 cells. Scale bar = 10 μm.

(d) Quantification of H3.1/3.2 and H3K27me3 intensity mentioned in (c). Mann-Whitney U test, error bar represents median with interquartile range. ‘n’ in X-axis represents number of cells analyzed per condition.

(e) Immunofluorescence staining of H3.1/3.2 and H3K27me3 in NUP210 knockdown MDA-MB-231 cells. Scale bar = 10 μm.

(f) Quantification of H3.1/3.2 and H3K27me3 intensity mentioned in (e). Mann-Whitney U test, error bar represents median with interquartile range. ‘n’ in X-axis represents number of cells analyzed per condition.

Supplementary Fig. 6

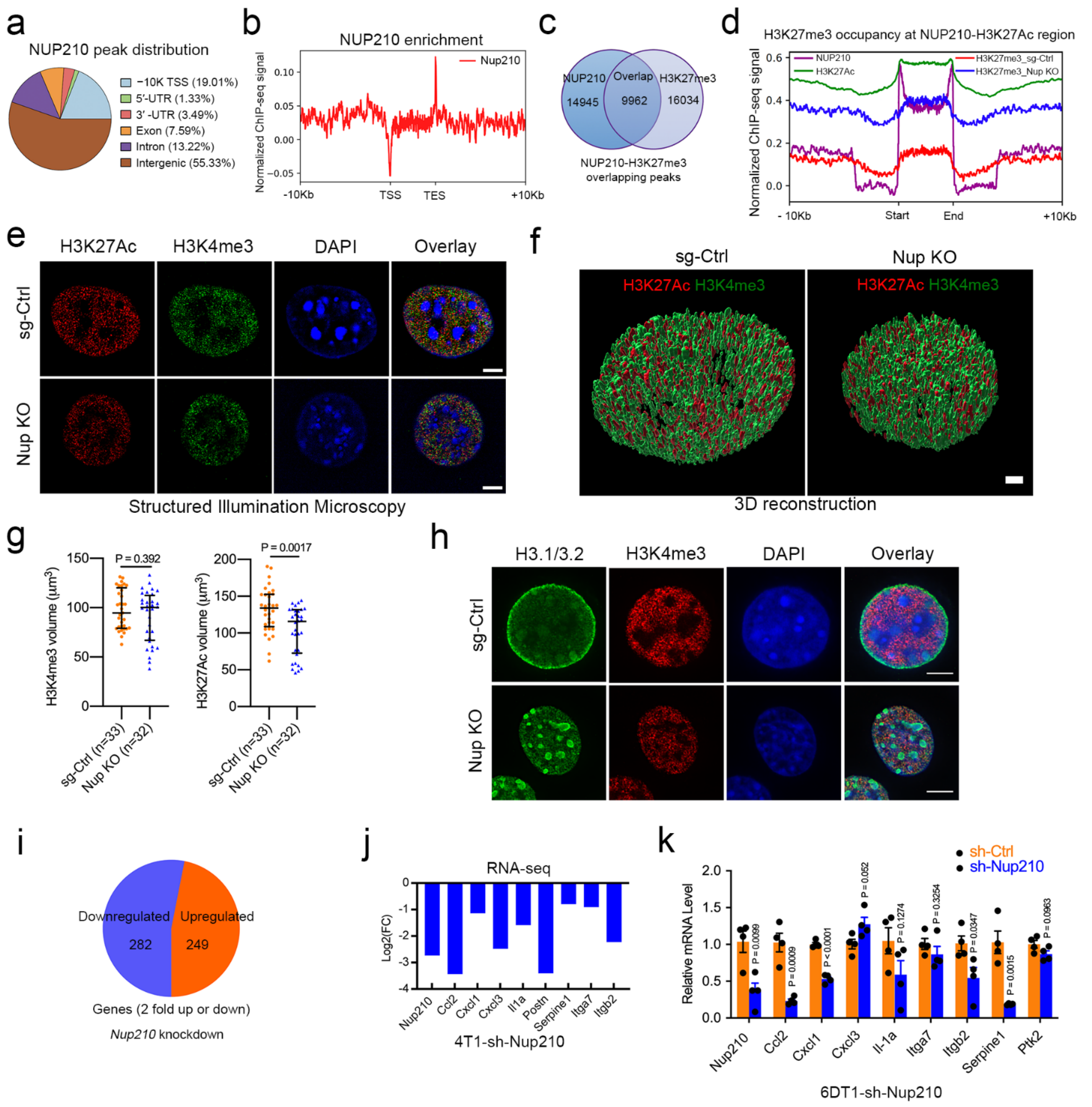

Supplementary Fig. 6: Related to Figure 5.

(a) Pie chart of NUP210 ChIP-seq peak distribution.

(b) ChIP-seq profile of NUP210 enrichment. TSS = Transcriptional start site, TES = Transcriptional end site.

(c) Overlap of NUP210 and H3K27me3 peaks.

(d) Occupancy of H3K27me3 peaks within the NUP210-H3K27Ac enhancer overlap region in sg-Ctrl and *Nup210* KO cells.

(e) Structured Illumination Microscopy images of H3K27Ac and H3K4me3 distribution in *Nup210* KO 4T1 cells. Scale bar = 5 μm.

(f) 3D reconstruction of H3K27Ac and H3K4me3 volume distribution in *Nup210* KO 4T1 cells. Scale bar = 2 μm.

(g) Quantification of H3K27Ac and H3K4me3 volume in *Nup210* KO 4T1 cells. Mann-Whitney U test, median with interquartile range. 'n' in X-axis represents number of cells analyzed per condition.

(h) Representative images of H3.1/3.2 and H3K4me3 distribution in *Nup210* KO 4T1 cells. Scale bar = 5 μm.

(i) RNA-seq analysis of up- or downregulated genes upon *Nup210* KD.

(j) Top downregulated genes from the RNA-seq of 4T1 *Nup210* KD (sh # 4) cells. FC=fold change.

(k) qRT-PCR analysis on *Nup210* KD 6DT1 cells. Multiple two tailed t-test, mean ± s.e.m, n = 4 biological replicates.

Supplementary Fig. 7

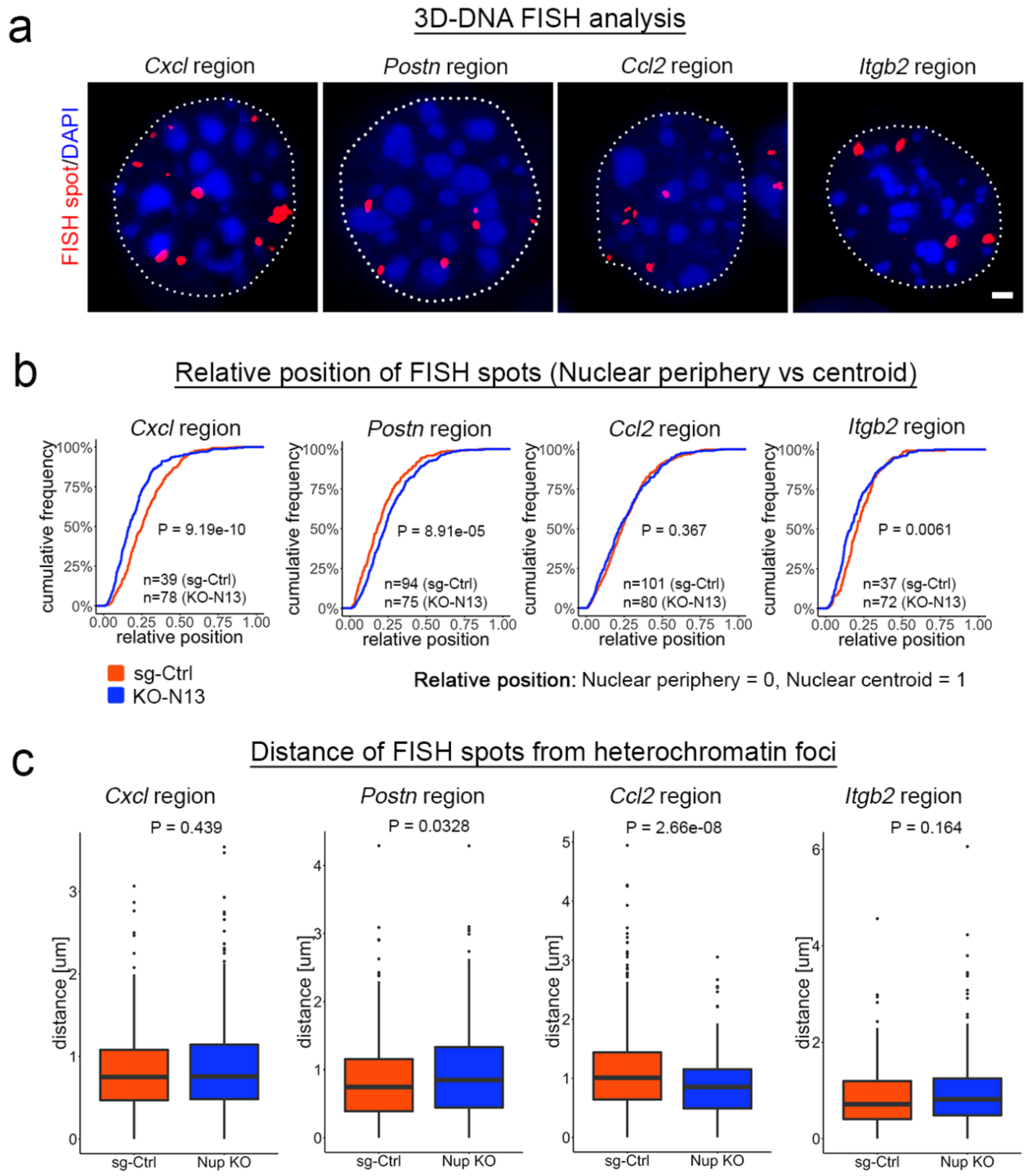

Supplementary Fig. 7: NUP210 loss is associated with differential repositioning mechanosensitive gene loci.

(a) Representative images of 3D-DNA FISH of NUP210-regulated gene loci within the nucleus. Scale bar = 2 μm.

(b) Cumulative distribution of FISH spots in nuclear periphery vs nuclear centroid in sg-Ctrl and *Nup210* KO 4T1 cells.

(c) Minimum distance of FISH spots from DAPI-stained heterochromatin foci in sg-Ctrl and *Nup210* KO 4T1 cell nuclei stated in (b). Mann-Whitney U test, the box extends from 25th to 75th percentile, whiskers extend from smallest values to the largest values, horizontal line represents median.

Supplementary Fig. 8

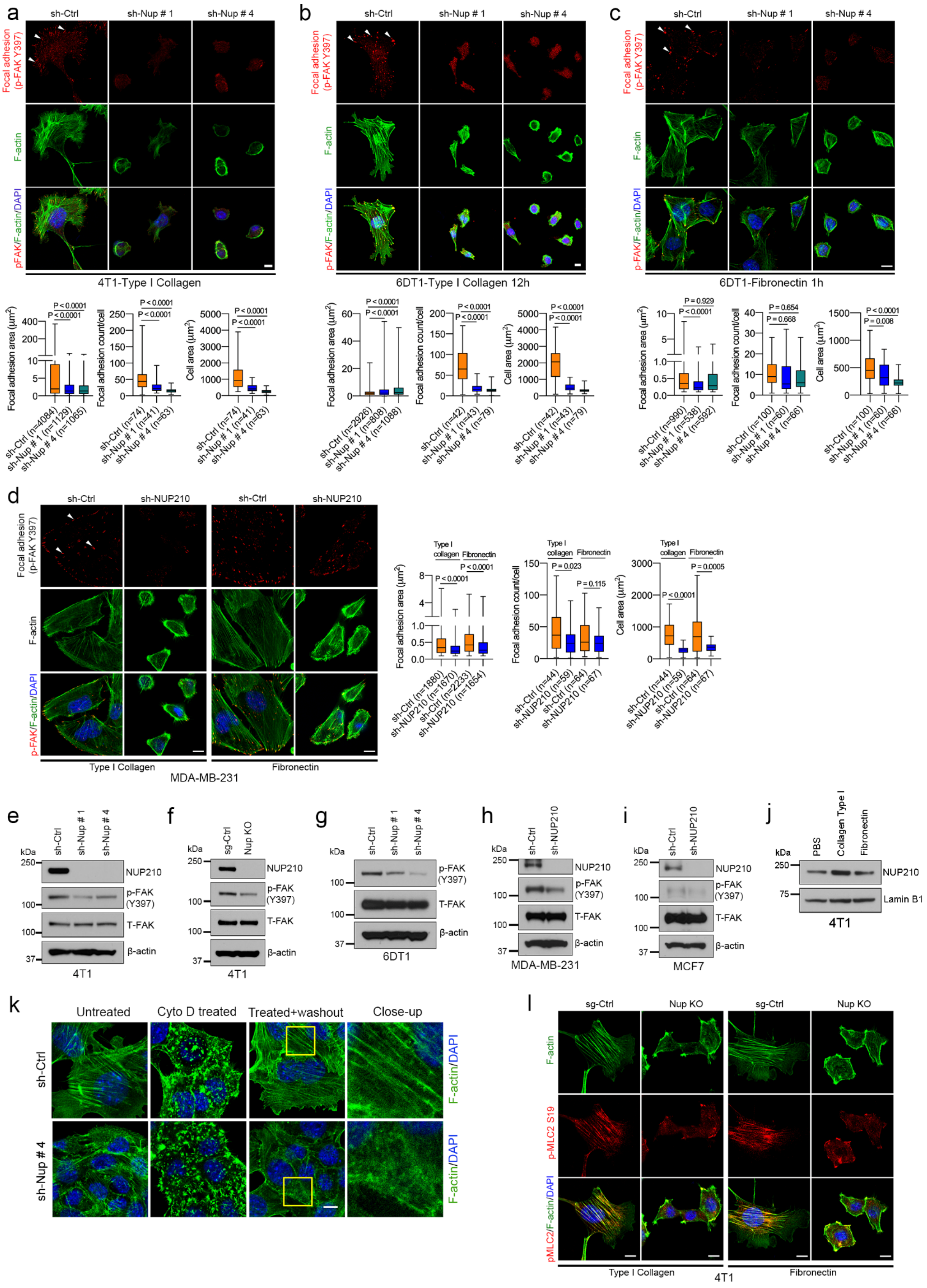

Supplementary Fig. 8: Related to Figure 6 and 7.

(a) (Top) Immunostaining of p-FAK Y397 and F-actin in *Nup210* KD 4T1 cells grown on Type I collagen. (Bottom) Quantification of focal adhesion (area, count) and cell spreading. ANOVA with Tukey's multiple comparison test. The box extends from 25th to 75th percentile, whiskers extend from smallest values to the largest values, horizontal line represents median. Scale bar = 10 μm.

(b) (Top) Immunostaining of p-FAK Y397 and F-actin in *Nup210* KD 6DT1 cells grown on Type I collagen. (Bottom) Quantification of focal adhesion (area, count) and cell spreading. ANOVA with Tukey's multiple comparison test. The box extends from 25th to 75th percentile, whiskers extend from smallest values to the largest values, horizontal line represents median. Scale bar = 10 μm.

(c) (Top) Immunostaining of p-FAK Y397 and F-actin in *Nup210* KD 6DT1 cells grown on fibronectin. (Bottom) Quantification of focal adhesion (area, count) and cell spreading. ANOVA with Tukey's multiple comparison test. The box extends from 25th to 75th percentile, whiskers extend from smallest values to the largest values, horizontal line represents median. Scale bar = 10 μm.

(d) (Left) Immunostaining of p-FAK Y397 and F-actin in *NUP210* KD MDA-MB-231 cells grown on Type I collagen or fibronectin. (Right) Quantification of focal adhesion and cell spreading. ANOVA with Tukey's multiple comparison test. The box extends from 25th to 75th percentile, whiskers extend from smallest values to the largest values, horizontal line represents median. Scale bar = 10 μm.

(e) Western blot of total FAK (T-FAK) and p-FAK (Y397) levels in *Nup210* KD 4T1, (f) *Nup210* KO 4T1, (g) *Nup210* KD 6DT1, (h) *NUP210* KD MDA-MB-231 and (i) *NUP210* KD MCF7 cells.

(j) Western blot of NUP210 and Lamin B1 on 4T1 cells grown on either Type I collagen or fibronectin.

(k) Representative images of F-actin stress fibers (phalloidin) after treatment and washout of cytochalasin D in sh-Ctrl and *Nup210* KD 4T1 cells. Scale bar = 10 μm.

(l) Representative images showing p-MLC2-S19 and F-actin staining on *Nup210* KO 4T1 cells grown on either Type I collagen or fibronectin. Scale bar = 10 μm.

Supplementary Fig. 9

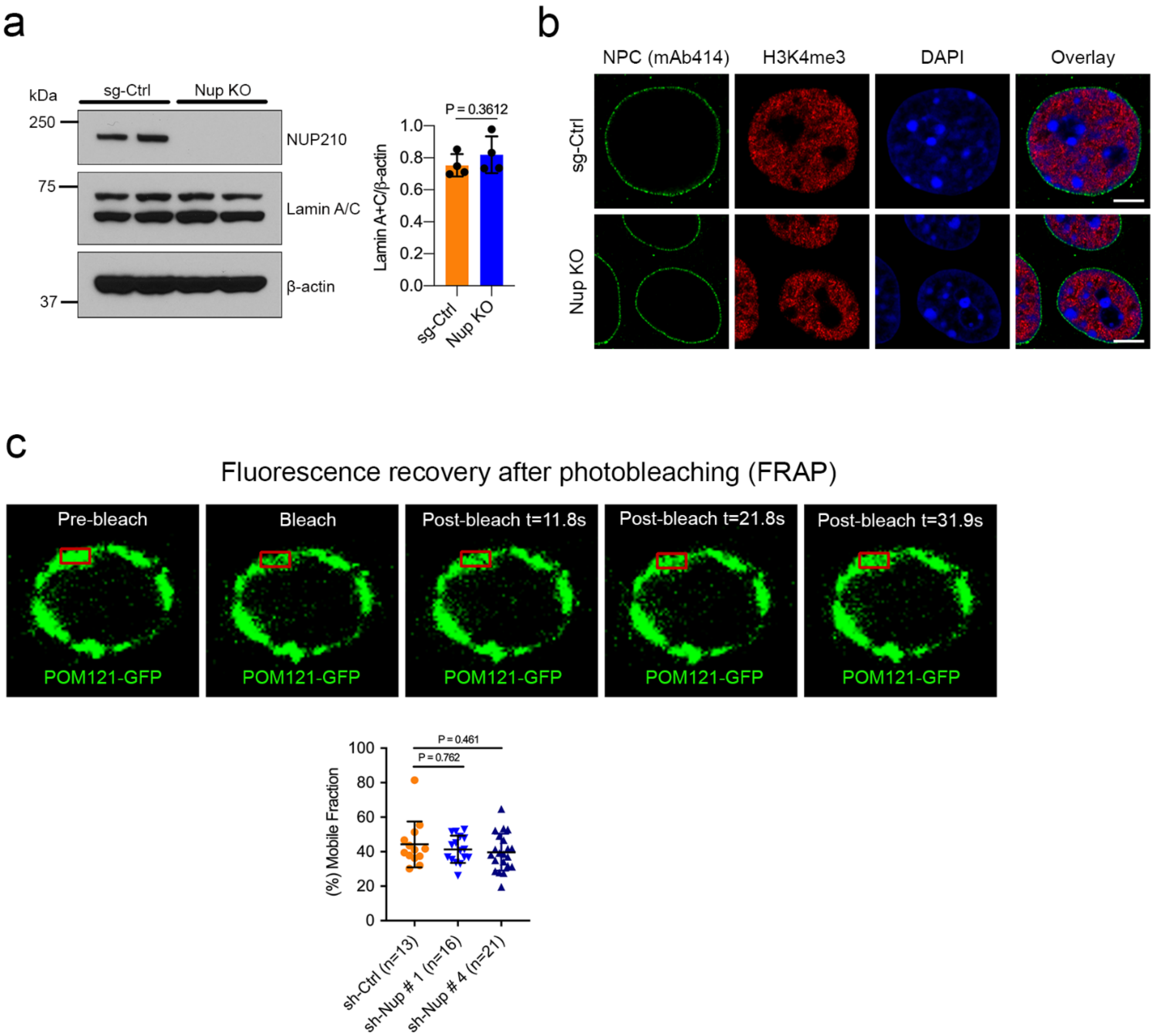

Supplementary Fig. 9: Effect of *Nup210* KO on Lamin A/C and on the dynamic distribution of nuclear pore structure.

(a) Representative western blot showing the protein level of Lamin A/C (Left) and densitometry quantification of intensity normalized to β-actin (right). Two tailed t-test, mean ± s.d. n = 4 biological replicates.

(b) Representative immunofluorescence images of the nuclear pore complex (stained using mAb414 antibody) and H3K4me3 in *Nup210* KO 4T1 cells. Scale bar = 5 μm.

(c) (Top) Fluorescence Recovery after Photobleaching (FRAP) analysis showing the dynamic distribution of GFP-tagged nuclear pore protein, POM121 in *Nup210* KD 4T1 cells. (Bottom) Quantification of POM121-

GFP mobile fraction. ANOVA with Tukey's multiple comparison test, mean ± s.d. 'n' in X-axis represents number of cells analyzed per condition.

Supplementary Fig. 10

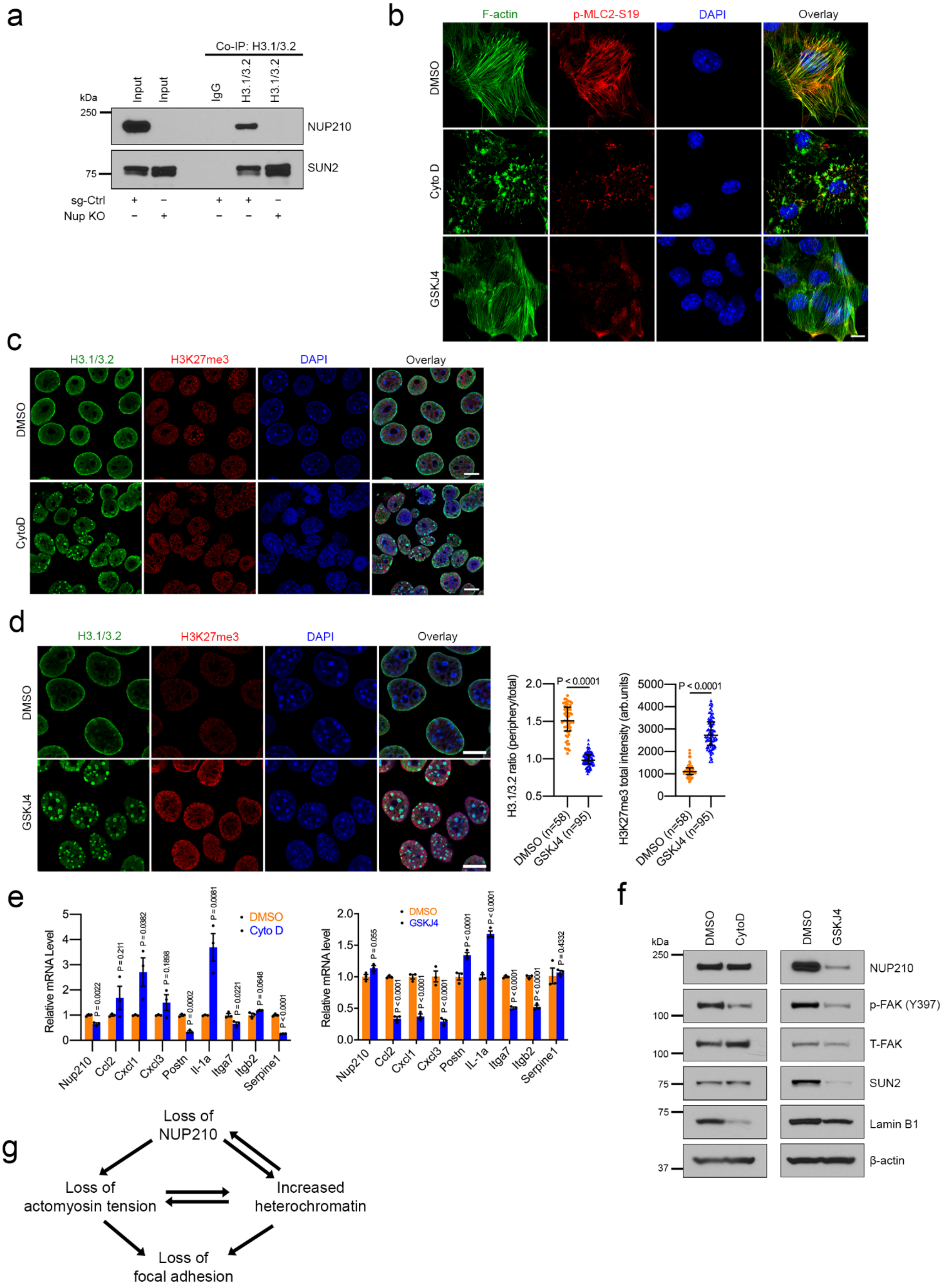

Supplementary Fig. 10: Related to Figure 8.

(a) Co-IP showing the interaction of H3.1/3.2 with NUP210 and SUN2 in *Nup210* KO 4T1 cells.

(b) Representative immunofluorescence images of F-actin and p-MLC2-S19 staining in Cytochalasin D or GSKJ4-treated 4T1 cells. Scale bar = 10 μm.

(c) Representative immunofluorescence images of H3.1/3.2 and H3K27me3 staining in Cytochalasin D-treated 4T1 cells. Scale bar = 10 μm.

(d) (Left) Representative immunofluorescence images of H3.1/3.2 and H3K27me3 staining in GSKJ4-treated 4T1 cells. (Right) Quantification of intensity distribution. Mann-Whitney U test, error bar represents median with interquartile range. 'n' in X-axis represents number of cells analyzed per condition. Scale bar = 10 μm.

(e) (Left) qRT-PCR of NUP210-regulated genes in Cytochalasin D-treated and (right) GSKJ4-treated 4T1 cells. Multiple two tailed t-test, mean ± s.e.m. n = 3 biological replicates.

(f) Western blot of NUP210, p-FAK Y397, T-FAK, SUN2 and Lamin B1 in either Cytochalasin D- or GSKJ4-treated 4T1 cells.

(g) Model depicting the feedback loop among the loss of NUP210, actomyosin tension, focal adhesion and heterochromatin regulation.

Supplementary Table 1: List of Reagents and oligonucleotides:

Reagents or resource	Company/source	Catalog
**Chemicals and other Reagents**		
DMEM	Gibco	Cat# 10313021
Fetal Bovine Serum	Gemini Bioproducts	Cat# 100-106
Penicillin:Streptomycin	Gemini Bioproducts	Cat# 400-100
L-Glutamine 200 mM	Gibco	Cat# 25030-081
0.25% Trypsin-EDTA (1X)	Gibco	Cat# 25200-056
Puromycin dihydrochloride	Sigma	Cat# P9620
Blasticidin	Gibco	Cat# R210-01
Benzonase Nuclease	Millipore	Cat# 70664
TriPure Isolation Reagent	Sigma	Cat# 11667165001
Collagen I, Rat Tail	Gibco	Cat# A10483-01
Vectashield antifade mounting medium with DAPI	Vector Laboratories	Cat# H-1200
RNeasy Mini Kit	Qiagen	Cat# 74106
Rnase-Free Dnase Set	Qiagen	Cat# 79254
DyLight 594 Labeled Anti-Digoxigenin (DIG)	Vector Laboratories	Cat# DI-7594
Fibronectin from bovine plasma	Sigma	Cat# F1141
GSK126	Selleckchem	Cat# S7061
GSKJ4	Selleckchem	Cat# S7070
FAK inhibitor (PND-1186)	Selleckchem	Cat# S7653
Leptomycin B	Cell Signaling Tech	Cat# 9676
Cytochalasin D	Sigma	Cat# C8273
Nuclear Extract Kit	Active Motif	Cat# 40010
Lab-Tek 2-well chambered glass cover slide	Thermo Fisher Scientific	Cat# 177380
Ibidi μ slide 4 well	Ibidi Inc.	Cat# 80426
Ibidi μ slide 8 well	Ibidi Inc.	Cat# 80826
Hoechst 33342	Thermo Fisher Scientific	Cat# 62249
Corning 96 Well Polystyrene Microplate	Corning	Cat# 3904
BioCoat Matrigel Invasion Chambers with 8.0 μm PET Membrane	Corning Inc.	Cat# 354480
FxCycle Violet Stain	Thermo Fisher Scientific	Cat# F10347
Ammonium bicarbonate	Sigma	Cat# A6141
Dynabead Protein G	Invitrogen	Cat# 10004D
NuPAGE LDS sample buffer	Invitrogen	Cat# NP0008
NuPAGE Sample Reducing Agent	Thermo Fisher Scientific	Cat# NP0009
NuPAGE 3-8% Tris-Acetate protein gel	Thermo Fisher Scientific	Cat# EA0375BOX
NuPAGE 4-12% Bis-Tris protein gel	Thermo Fisher Scientific	Cat# NP0321BOX
Novex 4-20% Tris-Glycine protein gel	Thermo Fisher Scientific	Cat# XP04200BOX
Immobilon-P PVDF Membrane	Millipore	Cat# IPVH07850
KOD Hot Start DNA Polymerase	Millipore	Cat# 71086
Quick Ligation Kit	New England Biolab	Cat# M2200
Gateway LR Clonase II Enzyme mix	Thermo Fisher Scientific	Cat# 11791020
SalI-HF	New England Biolab	Cat# R3138S
EcoRV-HF	New England Biolab	Cat# R3195S

KpnI	New England Biolab	Cat# R0142L
XhoI	New England Biolab	Cat# R0146L
T4 DNA ligase	New England Biolab	Cat# M0202S
X-tremeGENE 9 DNA transfection reagent	Roche	Cat# 06365809001
Nanojuice Transfection Reagent	Millipore	Cat# 71902
35 mm glass-bottom dish	MatTek	Cat# P35G-1.5-14-C
Target retrieval solution, citrate pH 6	Dako	Cat# S1699
Protein block, serum-free	Dako	Cat# X0909
Antibody diluent	Dako	Cat# S3022
Evision+System-HRP labelled polymer anti-Rabbit	Dako	Cat# K4003
DAB Substrate Kit	Vector lab	Cat# SK-4100
Prolong Glass Antifade Mountant	Invitrogen	Cat# P36982
Murine recombinant CCL2	Peprotech	Cat# 250-10
iScript cDNA Synthesis Kit	Bio-Rad	Cat# 1708890
FastStart Universal SYBR Green Master (Rox)	Roche	Cat# 4913850001
FosmidMax DNA Purification Kit	Epicentre	Cat# FMAX046
Atto550 NT Labeling Kit	Jena Bioscience	Cat# PP-305S-550
Digoxigenin NT Labeling Kit	Jena Bioscience	Cat# PP-310S-DIGX
Click-iT EdU Alexa Fluor 488 Flow Cytometry Assay Kit	Thermo Fisher Scientific	Cat# C-10425
ChIP-IT® Express Enzymatic Kit	Active Motif	Cat# 53009
TruSeq ChIP Library Preparation Kit	Illumina	Cat# IP-202-1012
TruSeq Stranded mRNA Library Prep Kit	Illumina	Cat# RS- 122-2201
Nuclear Complex Co-IP Kit	Active Motif	Cat# 54001
Pierce BCA Protein Assay Kit	Thermo Fisher Scientific	Cat# 23227
pENTR/D-TOPO Cloning Kit	Thermo Fisher Scientific	Cat# K240020
Dual-Luciferase Reporter Assay System	Promega	Cat# E1960
**Oligonucleotides**		
qRT-PCR primer for Nup210:<br>Forward: GGCGCACGATGTTCAGAA<br>Reverse: CACCACCAGGTCGAAATGGG	PrimerBank	https://pga.mgh.harvard.edu/primerbank/
qRT-PCR primer for Ppib:<br>Forward: GGAGATGGCACAGGAGGAAAGAG<br>Reverse: TGTGAGCCATTGGTGTCTTTGC	This paper	N/A
qRT-PCR primer for Gapdh:<br>Forward: AGGTCGGTGTGAACGGATTTG<br>Reverse: TGTAGACCATGTAGTTGAGGTCA	PrimerBank	https://pga.mgh.harvard.edu/primerbank/
qRT-PCR primer for Ccl2:<br>Forward: ATTAAAAACCTGGATCGGAACCAA<br>Reverse: GCATTAGCTTCAGATTTACGGGTC	This paper	N/A
qRT-PCR primer for Cxcl1:<br>Forward: CTGGGATTCACCTCAAGAACATC<br>Reverse: CAGGGTCAAGGCAAGCCTC	PrimerBank	https://pga.mgh.harvard.edu/primerbank/
qRT-PCR primer for Cxcl3:<br>Forward: CCCAGACAGAAGTCATAGCCACT<br>Reverse: TTCATCATGGTGAGGGGCTTC	This paper	N/A
qRT-PCR primer for Postn:<br>Forward: AATGCTGCCCTGGCTATATGAG<br>Reverse: GTAGTGGCTCCCACAATGCC	This paper	N/A

qRT-PCR primer for Il1a (IL-1a):<br>Forward: CGAAGACTACAGTTCTGCCATT<br>Reverse: GACGTTTCAGAGGTTCTCAGAG	PrimerBank	https://pga.mgh.harvard.edu/primerbank/
qRT-PCR primer for Itga7:<br>Forward: CTGCTGTGGAAGCTGGGATTC<br>Reverse: CTCCTCCTTGAACTGCTGTCG	PrimerBank	https://pga.mgh.harvard.edu/primerbank/
qRT-PCR primer for Itgb2:<br>Forward: CAGGAATGCACCAAGTACAAAGT<br>Reverse: CCTGGTCCAGTGAAGTTCAGC	PrimerBank	https://pga.mgh.harvard.edu/primerbank/
qRT-PCR primer for Serpine1:<br>Forward: TTCAGCCCTTGCTTGCCTC<br>Reverse: ACACTTTTACTCCGAAGTCGGT	PrimerBank	https://pga.mgh.harvard.edu/primerbank/
qRT-PCR primer for Ier3:<br>Forward: GCTCTGGTCCCGAAATTTTCA<br>Reverse: AGATGATGGCGAACAGGAGAA	PrimerBank	https://pga.mgh.harvard.edu/primerbank/
qRT-PCR primer for Brd4-SF<br>Forward: ATCCCCTGGGGTGGATTAGT<br>Reverse: AGAGGACCCCAGATGACCAG	This paper	N/A
qRT-PCR primer for Yap1<br>Forward: TACTGATGCAGGTACTGCGG<br>Reverse: TCAGGGATCTCAAAGGAGGAC	PrimerBank	https://pga.mgh.harvard.edu/primerbank/
qRT-PCR primer for Mrtf-a<br>Forward: CTGGTGAGCCAAGGGATCAT<br>Reverse: TCAGCCGAGGTCTCTTCCAG	This paper	N/A
qRT-PCR primer for Ptk2 (FAK)<br>Forward: GAGTACGTCCCTATGGTGAAGG<br>Reverse: CTCGATCTCTCGATGAGTGCT	PrimerBank	https://pga.mgh.harvard.edu/primerbank/
Nup210 targeting sgRNA cloning primer:<br>sgRNA-sense:<br>CACCGCGACACCATCCTAGTGTCT<br>sgRNA-antisense:<br>AAACAGACACTAGGATGGTGTCGC	This paper	N/A
sgRNA non-targeting control sequence:<br>CCATATCGGGGCGAGACATG	Ji Luo Lab (NCI)	N/A
Nup210 KO sgRNA sequencing primer:<br>Forward: TGTGTGCCTGCTTCAGGATAAG		
sgRNA targeting Nup210 promoter CTCF binding site:<br>sg-sense-Top:<br>ACCGCCAAAGCCACACTTCCCCAG<br>sg-sense-Bottom:<br>AAACCTGGGGAAGTGTGGCTTTGG<br>sg-antisense-Top:<br>ACCGGAGCGCTCCACACTGTCCTA<br>sg-antisense-Bottom:<br>AAACTAGGACAGTGTGGAGCGCTC	This paper	N/A
Nup210 promoter sgRNA sequencing primer:<br>Forward: CTTGATGTACTGCCAAGTGGGC	Chiang et al. 2016	https://www.nature.com/articles/srep24356
Primer for Histone H3.1 cloning into pENTR/D-TOPO vector<br>Histone H3.1 Forward:<br>CACCATGGACTACAAAGACCATGACGG<br>Histone H3.1 Reverse:<br>TGCCCTTTCCCCACGGAT	This paper	N/A

Histone H3.3 Reverse:<br>AGCTCTCTCCCCGTATCC		
Nup210 promoter cloning primer:<br>KpnI-Nup210-prom-Forward:<br>TGCTTA- GGTACC-<br>TTGTCCTGCTGATGGATTGAT<br>XhoI-Nup210-prom-Reverse:<br>TAAGCA-CTCGAG-<br>AGCAGCACCTTGGGAATGTTA	This paper	N/A
Primer for cloning mouse FAK into pENTR/D-TOPO vector<br>FAK-Forward:<br>CACCATGGCAGCTGCTTATCTTGACCC<br>FAK-Reverse:<br>GTGTGGCCGTGTCTGCCC	This paper	N/A
Primer for cloning mouse Ccl2 into pENTR/D-TOPO vector<br>Ccl2-Forward:<br>CACCATGCAGGTCCCTGTCATGCTTC<br>Ccl2-Reverse:<br>GTTCACTGTCACACTGGTCACTCC	This paper	N/A
ChIP qRT-PCR primer for CTCF and H3K27Ac enrichment on Nup210 promoter:<br>Forward: CTGCTGATGGATTGATCCCGG<br>Reverse: GTGTGGAGCGCTCACTGATT	This paper	N/A
**Plasmids:**		
Plasmid: pxPAX2	Addgene	Cat# 12260
Plasmid: pMD2.G	Addgene	Cat# 12259
Plasmid: lentiGuide-puro	Addgene	Cat# 52963
Plasmid: Ad (RGD)-GFP-Cas9	Vector Biolab	Cat# 1903
Plasmid: AIO-GFP	Addgene	Cat# 74119
Plasmid: pCMV6-mNup210	Origene Technologies	Cat# MR219004
Plasmid: pDest-658-mPol2-mNup210-Myc	This Study	N/A
Plasmid: pENTR/D-TOPO	Thermo Fisher Scientific	Cat# K240020
Plasmid: POM121-GFP	Genecopoeia	Cat# EX-Mm22993-M98
Plasmid: pDest-659-3X-Flag-Histone H3.1	This Study	N/A
Plasmid: pMXs-puro-EGFP-FAK	Addgene	Cat# 38194
Plasmid: pDest-659-FAK-myc	This Study	N/A
Plasmid: pDest-659-Ccl2-myc	This Study	N/A
BAC clone: RP23-374O6	CHORI	N/A
BAC clone: RP23-480C1	CHORI	N/A
BAC clone: RP23-99N1	CHORI	N/A
BAC clone: RP23-166E21	CHORI	N/A
