# Supplementary material for: Nuclear pore protein NUP210 depletion suppresses metastasis through heterochromatin-mediated disruption of tumor cell mechanical response
Source: Nat Commun. 2021 Dec 13;12:7216. doi: 10.1038/s41467-021-27451-w (PMC8669001; doi:10.1038/s41467-021-27451-w)
Supplement: Supplementary file 3 — Description of Additional Supplementary Files [file 41467_2021_27451_MOESM3_ESM.pdf]

Description of Additional Supplementary Files

Supplementary Movie 1: 3D reconstruction of Z-stack images from H3.1/3.2 and H3K9me3 staining in 4T1 sg-Ctrl cells.

Supplementary Movie 2: 3D reconstruction of Z-stack images from H3.1/3.2 and H3K9me3 staining in 4T1 *Nup210* KO cells.

Supplementary Movie 3: 3D reconstruction of Z-stack images from H3K27Ac (enhancer) and H3K4me3 (promoter) staining in 4T1 sg-Ctrl cells.

Supplementary Movie 4: 3D reconstruction of Z-stack images from H3K27Ac (enhancer) and H3K4me3 (promoter) staining in 4T1 *Nup210* KO cells.

Supplementary Movie 5: Live cell imaging of F-tractin (red) and MLC2 (Cyan) reporter in 6DT1 sh-Ctrl cells after treatment with Cytochalasin D.

Supplementary Movie 6: Live cell imaging of F-tractin (red) and MLC2 (Cyan) reporter in 6DT1 sh-*Nup210* cells after treatment with Cytochalasin D.

Supplementary Movie 7: Live cell tracking of cell migration (measured by Hoechst staining of nuclei) in 4T1 sg-Ctrl cells.

Supplementary Movie 8: Live cell tracking of cell migration (measured by Hoechst staining of nuclei) in 4T1 *Nup210* KO-N9 cells.

Supplementary Movie 9: Live cell tracking of cell migration (measured by Hoechst staining of nuclei) in 4T1 *Nup210* KO-N13 cells.

Supplementary Movie 10: 3D reconstruction of Z-stack images from Lamin B1 (red), Lamin A/C (green) and heterochromatin foci (DAPI, cyan) staining in 4T1 sg-Ctrl cells.

Supplementary Movie 11: 3D reconstruction of Z-stack images from Lamin B1 (red), Lamin A/C (green) and heterochromatin foci (DAPI, cyan) staining in 4T1 *Nup210* KO cells.
