# Supplementary material for: Nuclear pore protein NUP210 depletion suppresses metastasis through heterochromatin-mediated disruption of tumor cell mechanical response
Source: Nat Commun. 2021 Dec 13;12:7216. doi: 10.1038/s41467-021-27451-w (PMC8669001; doi:10.1038/s41467-021-27451-w)
Supplement: Supplementary file 15 — Reporting Summary [file 41467_2021_27451_MOESM15_ESM.pdf]

nature research

Corresponding author(s): Ruhul Amin; Kent W. Hunter

Last updated by author(s): Nov 15, 2021

Reporting Summary

Nature Research wishes to improve the reproducibility of the work that we publish. This form provides structure for consistency and transparency in reporting. For further information on Nature Research policies, see Authors & Referees and the Editorial Policy Checklist.

Statistics

For all statistical analyses, confirm that the following items are present in the figure legend, table legend, main text, or Methods section.

n/a	Confirmed	
☐	☒	The exact sample size (*n*) for each experimental group/condition, given as a discrete number and unit of measurement
☐	☒	A statement on whether measurements were taken from distinct samples or whether the same sample was measured repeatedly
☐	☒	The statistical test(s) used AND whether they are one- or two-sided<br>*Only common tests should be described solely by name; describe more complex techniques in the Methods section.*
☒	☐	A description of all covariates tested
☐	☒	A description of any assumptions or corrections, such as tests of normality and adjustment for multiple comparisons
☐	☒	A full description of the statistical parameters including central tendency (e.g. means) or other basic estimates (e.g. regression coefficient) AND variation (e.g. standard deviation) or associated estimates of uncertainty (e.g. confidence intervals)
☐	☒	For null hypothesis testing, the test statistic (e.g. *F*, *t*, *r*) with confidence intervals, effect sizes, degrees of freedom and *P* value noted<br>*Give P values as exact values whenever suitable.*
☒	☐	For Bayesian analysis, information on the choice of priors and Markov chain Monte Carlo settings
☒	☐	For hierarchical and complex designs, identification of the appropriate level for tests and full reporting of outcomes
☒	☐	Estimates of effect sizes (e.g. Cohen's *d*, Pearson's *r*), indicating how they were calculated

Our web collection on statistics for biologists contains articles on many of the points above.

Software and code

Policy information about availability of computer code

Data collection	Microscopy: ZEN Black V2.3, Nikon NIS Element V5.11, Image-Pro Premier 3D<br>Flow Cytometry: BD FACSCanto II<br>Reporter assay: GLOMAX V1.9.3 connected to luminometer.<br>Live cell imaging: Nikon Eclipse Ti2 Microscope software
Data analysis	Statistical analysis: Graph Pad Prism V8, RStudio.<br>Microscopy image data analysis: ImageJ, Fiji 2, ZEN Blue V2, ZEN Black V2.3, Nikon NIS Element V5.11, I, Image-Pro Premier 3D, Imaris V9.7.1.<br>Genomics data analysis: Sicer V1.1, deepTools2, bedtools, R computing environment, Perl, ChIPSeeker, Integrative Genomic Viewer (IGV), Partek Flow, Trimomatic software, Bowtie2.<br>RNA-seq data analysis: Partek Flow.<br>Gene Ontology Analysis: PANTHER classification system (Mi et al. 2019. Nat. Protoc).<br>Flow cytometry data analysis: FlowJo V10.<br>Automated live cell imaging analysis: MATLAB script (Cappell et al. 2016. Cell).

For manuscripts utilizing custom algorithms or software that are central to the research but not yet described in published literature, software must be made available to editors/reviewers. We strongly encourage code deposition in a community repository (e.g. GitHub). See the Nature Research guidelines for submitting code & software for further information.

Data

Policy information about availability of data

All manuscripts must include a data availability statement. This statement should provide the following information, where applicable:

- Accession codes, unique identifiers, or web links for publicly available datasets
- A list of figures that have associated raw data
- A description of any restrictions on data availability

Gene expression (RNA-seq) and ChIP-seq data have been deposited in Gene Expression Omnibus (GEO) database under the accession number GSE146591. All the source data underlying figures and unprocessed western blot images are provided as a source data file. Supplementary movie files (1-11) are provided as separate supplementary data files. All the other data are available within the article and its Supplementary Information. A reporting summary of this article is available as a supplementary information file.
Following publicly available database was used:
Sanger Mouse Genome Project Database (https://www.sanger.ac.uk/data/mouse-genomes-project/)
cBioportal Database (https://www.cbioportal.org/)
Km-plotter Database (https://kmplot.com/analysis/)
Gene Expression Omnibus Database (https://www.ncbi.nlm.nih.gov/geo/)

Field-specific reporting

Please select the one below that is the best fit for your research. If you are not sure, read the appropriate sections before making your selection.

☒ Life sciences ☐ Behavioural & social sciences ☐ Ecological, evolutionary & environmental sciences

For a reference copy of the document with all sections, see nature.com/documents/nr-reporting-summary-flat.pdf

Life sciences study design

All studies must disclose on these points even when the disclosure is negative.

Sample size	No statistical tests were performed to pre-determine sample size. Sample sizes for the spontaneous metastasis assays (10 mice per experimental condition) were selected due to more than 20 years previous experience demonstrating that this is the minimum number of animals necessary to achieve statistically significant and reproducible results (e.g. Ha et al. 2016. Plos Genet. PMID: 27656887; Faraji et al. 2016. Plos Genet. PMID: 26807845, Park et al. 2005 Nat. Genet. PMID:16142231). For experiments involving cell cultures, at least 3 biological replicates per condition was used to enable statistical analysis.
Data exclusions	No data were excluded from the analysis.
Replication	Experiments have been repeated at least duplicate or triplicates. For studies involving mice (metastasis assay), experiment was performed once for each orthopic transplantation models to limit the use of animals. We have used 3 different immunocompetent orthotopic transplantation model of metastasis and 2 different mice background to validate the robustness of key results in different biological systems. All the attempts at replication of experimental conditions were successful.
Randomization	For animal studies, mice were randomly chosen into each experimental groups. For all other samples used in this study, including cultured cells, were allocated randomly to each condition.
Blinding	Investigators were not blinded during allocating animal experiment because the investigators needed to know what cell type they will be injecting and they had to culture and process the cells for mouse injection by themselves. Assessment of immunohistochemistry result was performed by independent researcher in the blinded fashion. In many applications (Mass spectrometry analysis, Accessible chromatin analysis, ChIA-PET seq, RNA-seq, ChIP-seq, high-resolution microscopy, DNA fluoresence in situ hybridization etc), acquisition of the data was performed by expert technicians without the knowledge of the experimental groups in a blinded fashion.

Reporting for specific materials, systems and methods

We require information from authors about some types of materials, experimental systems and methods used in many studies. Here, indicate whether each material, system or method listed is relevant to your study. If you are not sure if a list item applies to your research, read the appropriate section before selecting a response.

Materials & experimental systems

n/a	Involved in the study
	☒ Antibodies
	☒ Eukaryotic cell lines
☒	☐ Palaeontology
	☒ Animals and other organisms
☒	☐ Human research participants
☒	☐ Clinical data

Methods

n/a	Involved in the study
	☒ ChIP-seq
	☒ Flow cytometry
☒	☐ MRI-based neuroimaging

Antibodies

Antibodies used

Mouse anti-beta-ACTIN, Abcam, Cat# ab6276, 1:10,000 WB
Rabbit anti-beta-tubulin, Cell Signaling Tech, Cat# 86298, 1:1000 WB
Rabbit anti-NUP210, Bethyl Laboratories, Cat# A301-795A, 1:500 WB, 2 ug IP, 2 ug ChIP
Rabbit anti-NUP210, Atlas antibodies, Cat# HPA066888, 1:100 for IHC
Rabbit anti-SUN1, Abcam, Cat# ab103021, 1: 500 for WB, 1:100 for IF
Rabbit anti-SUN2, Abcam, Cat# ab124916, 1:1,000 WB
Mouse anti-SUN2, Millipore, Cat# MABT880, 1:100 for IF
Rabbit anti-FAK,Abcam, Cat# ab40794, 1:5,000 WB, 1:100 for IF
Rabbit anti-p-FAK (Y397), Abcam Cat# ab39967, 1:5,000 WB, 1:100 IF
Rabbit anti-p-FAK (Y397), Thermofisher Scientific, Cat# 700255, 1:500 for Tissue IF
Mouse anti-CCL2, Proteintech, Cat# 66272-I-Ig, 1:500 for WB
Mouse anti-Nuclear pore complex (mAb414), Abcam Cat# ab24609, 1:1000 IF, 2 ug ChIP
Rabbit anti-Lamin B1, Abcam Cat# ab16048, 1:5,000 WB
Mouse anti-Lamin A/C, Abcam, Cat# ab8984, 1:1000 for WB, 1:500 for IF
Rabbit anti-Nucleolin, Abcam Cat# ab22758, 1:500 IF
Mouse anti-Myc-Tag, Cell Signaling Tech, Cat# 2276, 1:1,000 WB, I:500 IF, 2 ug IP
Rabbit anti-Myc-Tag, Cell Signaling Tech, Cat# 2272, 1:100 IF
Rabbit anti-V5-Tag, Cell Signaling Tech, Cat# 13202, 1:1000 for WB
Mouse anti-V5-Tag, Cell Signaling Tech, Cat# 80076, 1:1000 for WB
Rabbit anti-p-MLC2 (S19), Cell Signaling Tech, Cat# 3671, 1:100 for IF
Rabbit anti-RRP1B, Millipore, Cat# HPA020324, 1:1000 for WB
Rabbit anti-YAP, Cell Signaling Tech, Cat#14074, 1:1000 for WB
Rabbit anti-MRTF-A, Cell Signaling Tech, Cat#14760, 1:1000 for WB
Mouse anti-H3.1/3.2, Active Motif Cat# 61629, 1:1000 WB, 1:1,000 IF, 2 ug IP
Rabbit anti-H3K27me3, Cell Signaling Tech Cat# 9733, 1:5,000 WB, 1:1,000 IF, 10 ul ChIP
Mouse anti-H3K27me3, Abcam Cat# ab6002, 1:100
Rabbit anti-H3K4me3, Millipore Cat# 04-754, 1:5,000, 10 ul ChIP
Rabbit anti-H3K9me3, Abcam Cat# ab8898, 1:5,000
Rabbit anti-EZH2, Cell Signaling Tech Cat# 5246, 2 ug IP
Mouse anti-EZH2, Cell Signaling Tech Cat# 3174, 1:1,000 WB
Rabbit anti-SUZ12, Cell Signaling Tech Cat# 3737, 1:1,000 WB
Rabbit anti-SUV39H1, Cell Signaling Tech Cat# 8729, 1:1,000 WB
Rabbit anti-CTCF, Cell Signaling Tech Cat# 2899, 10 ul ChIP
Rabbit anti-H3K27Ac, Cell Signaling Tech Cat# 8173, 10 ul ChIP
Anti-Rabbit Horseradish peroxidase (HRP), Cell Signaling Tech Cat# 7074, 1: 3,000
Anti-Mouse HRP, GE Healthcare Cat# NA931, 1:10,000 WB
Rabbit IgG Control, Cell Signaling Tech Cat# 3900, 2 ug IP, ChIP
Mouse IgG Control, Santa Cruz Biotech Cat# sc-2025, 2 ug IP, ChIP
Mouse anti-Pan-Keratin, Alexa Fluor 488 Cell Signaling Tech Cat# 4523, 1:25 FC, 1:100 for tissue IF
Mouse IgG1-Isotype Control Alexa Fluor 488 Cell Signaling Tech Cat# 4878, 1:25 FC
APC rat IgG2b, k Isotype Control, Biolegend Cat# 400612, 1:50 FC
Mouse FcR blocking reagent, Miltenyi Biotec Cat# 130-092-575, 1:10 FC
Mouse anti-CD45 APC clone 30F11, Miltenyi Biotec Cat# 130-102-544, 1:25 FC
Goat anti-Mouse Alexa Fluor 488, Invitrogen Cat# A11029, 1:200 IF
Goat anti-Rabbit Alexa Fluor 488, Invitrogen Cat# A11008, 1:200 IF
Goat anti-Rabbit Alexa Fluor 568, Invitrogen Cat# A11011, 1:200 IF
Goat anti-Rabbit Alexa Fluor 594, Invitrogen Cat# A11037, 1:200 IF
Goat anti-Mouse Alexa Fluor 594, Invitrogen Cat# A11032, 1:200 IF
Phalloidin Alexa Fluor 488, Invitrogen Cat# A12379, 1:250 IF

Validation

All the antibodies used in this study are commercially available and extensively validated by the company, us or others. Validation data is available in each of these company's website. In addition to the validation data of NUP210 antibody in it's manufacturer's (Bethyl Laboratories) website, we have confirmed the specificity of the NUP210 antibody in our lab using western blot, immunofluoresence in NUP210 knockdown/knockout human (MCF7, MDA-MB-231) and mouse (4T1, 6DT1) cell lines. We further checked the specificity of the NUP210 antibody using peptide competition assay and coimmunoprecipitation followed by mass spectrometry analysis.

Eukaryotic cell lines

Policy information about cell lines

Cell line source(s)	Murine 4T1, 4T07, 6DT1 and MVT1 cell lines have been received from Dr. Lalage Wakefield Lab (NCI). Human breast cancer cell lines MCF7 and MDA-MB-231 cell lines have been received from Dr. Geffrey E. Green Lab (NCI). 293 FT cells were purchased from Thermo Fisher Scientific.
Authentication	Cell lines were not authenticated in our laboratory.
Mycoplasma contamination	We have extensively checked our cell lines for Mycoplasma contamination using Mycoalert Mycoplasma detection kit (Lonza) and found negative.
Commonly misidentified lines (See ICLAC register)	None of the cell lines used in this study were found in the database of misidentified cell lines

Animals and other organisms

Policy information about studies involving animals; ARRIVE guidelines recommended for reporting animal research

Laboratory animals	~6 weeks old, female BALB/cJ (000651) and FVB/NJ (001800) mice were purchased from The Jackson Laboratory. Mice were housed in 12 hours dark/12 hours light cycle, ambient temperature and humidity condition.
Wild animals	The study did not involve any wild animal.
Field-collected samples	The study did not involve field-collected samples.
Ethics oversight	Usage of animals described in this study was performed under the animal study protocol LCBG-004 approved by the National Cancer Institute (NCI) at Bethesda Animal Use and Care Committee.

Note that full information on the approval of the study protocol must also be provided in the manuscript.

ChIP-seq

Data deposition

☒ Confirm that both raw and final processed data have been deposited in a public database such as GEO.

☒ Confirm that you have deposited or provided access to graph files (e.g. BED files) for the called peaks.

Data access links<br>*May remain private before publication.*	https://www.ncbi.nlm.nih.gov/geo/query/acc.cgi?acc=GSE146591
Files in database submission	Title:argeting nuclear pore protein, NUP210, reduces metastasis through heterochromatin-mediated silencing of mechanosensitive genes<br>Samples:<br>GSM4396319 H3K27me3_sg-Ctrl<br>GSM4396320 H3K27me3_KO-N13<br>GSM4396321 H3K4me3_sg-Ctrl<br>GSM4396322 H3K4me3_KO-N13<br>GSM4396323 NUP210_sg-Ctrl<br>GSM4396324 NPC_sg-Ctrl<br>GSM4396325 4T1_IgG_R_2 Control<br>GSM4396326 4T1_IgG_Rb Control<br>GSM4396327 4T1_IgG_Ms Control
Genome browser session (e.g. UCSC)	We viewed all of our data using Integrative Genomic Viewer (IGV) which is not a public browser website. We can provide the bigwig file of our data for viewing in IGV if needed.

Methodology

Replicates	ChIP-seq was performed on one replicate per condition. ChIP enrichment was normalized to negative control (IgG).
Sequencing depth	All ChIP-seq experiments were performed in single-end read, 75bp.<br>Total mapped reads (trimmed):<br>4T1-IgG-R1: 39,886,755<br>4T1-H3K4me3-sg-Ctrl: 36,701,153<br>4T1-H3K4me3-KO-N13: 48,155,331<br>4T1-H3K27me3-sg-Ctrl: 31,098,784<br>4T1-H3K27me3-KO-N13: 98,429,857<br>4T1-IgG-Rb-2: 46,666,013<br>4T1-NUP210: 45,662,706

	4T1-IgG-Ms: 72,593,455<br>4T1-IgG-NPC: 44,655,297
Antibodies	Antibodies used for ChIP are the same listed above.
Peak calling parameters	ChIP-seq data was analyzed using the Sicer 1.1 algorithm (Xu et al., 2014) with default parameters. For narrow peaks such as H3K4me3 enrichment, a window size of 200 was used. For broad peaks such as H3K27me3 and NUP210, a window size of 1000 was used.
Data quality	All the called peaks in this study from Sicer was at 5% FDR
Software	Sicer 1.1, deeptools2, ChIPSeeker 3.1, IGV 2.4.14

Flow Cytometry

Plots

Confirm that:

☒ The axis labels state the marker and fluorochrome used (e.g. CD4-FITC).

☒ The axis scales are clearly visible. Include numbers along axes only for bottom left plot of group (a 'group' is an analysis of identical markers).

☒ All plots are contour plots with outliers or pseudocolor plots.

☒ A numerical value for number of cells or percentage (with statistics) is provided.

Methodology

Sample preparation	Circulating tumor cell (CTC) analysis:<br>100,000 6DT1 cells with or without Nup210 knockdown were injected into the fourth mammary fat pad of FVB/NJ mice. Ten mice were used in each group and three mice were kept uninjected for use as healthy controls. One month after injection, mice were anesthetized with avertin injection. Through cardiac puncture, 600-1000 ul blood per mouse was collected in 50 ul of 0.5 M EDTA solution. An equal volume of blood was taken for red blood cell lysis using ACK lysis buffer. 100 ul of the peripheral blood lymphocyte (PBL) fraction was subjected to fixation with 2% paraformaldehyde for 15 min at room temperature. Cells were permeabilized with PBS containing 0.1% Triton X-100. Cells were vortexed briefly and kept at room temperature for 30 min. 0.5% BSA in PBS was added and cells were pelleted by centrifugation. Cells were then resuspended in ice-cold 50% methanol in PBS and incubated for 10 min on ice. 150,000 fixed cells were stained for CD45, a pan-lymphocyte marker, and pan-keratin, a tumor cell marker. Before staining with antibodies, cells were incubated with FcR Blocking Reagent (1:10 dilution; Miltenyi) for 10 min at 4 degree C. Cells were then stained with APC-conjugated CD45 (1:25 dilution; Miltenyi) and Alexa Fluor 488-conjugated pan-keratin (1:25 dilution, Cell Signaling Technology) antibodies for 10 min at 4 degree C. After washing with MACS buffer (PBS, 0.5% BSA, and 2 mM EDTA), cells were incubated with 1 ug/ml Hoechst 33342 (Thermo Fisher Scientific) for 5 min. Cells were then washed again with MACS buffer and resuspended in 200 ul buffer for analysis using a BD FACSCanto II flow cytometer. A CTC (CD45-/Cytokeratin+) gate was created based on the staining pattern of 6DT1 tumor cells in culture and primary tumor cells derived from 6DT1-injected mice.<br><br>Cell cycle analysis:<br>Cell cycle analysis was performed using the Click-iT EdU Alexa Fluor 488 Flow Cytometry Assay Kit (Thermo Fisher Scientific) and FxCycle Violet Stain (Thermo Fisher Scientific) according to manufacturer's instructions. Briefly, 4T1 cells were seeded onto 15 cm tissue culture dishes at a seeding density of 3x106 cells/dish. 24 h later, cells were pulsed with Click-iT EdU (5-ethynyl-2´-deoxyuridine) for 1 h. After harvesting the cells through trypsinization, cells were fixed with Click-iT fixative and permeabilized with saponin-based permeabilization agent. The Click-iT reaction was then performed for 30 min at room temperature. Cells were then washed with wash buffer and stained for DNA content analysis with FxCycle Violet, a DNA-selective dye. Finally, cell cycle analysis was performed using a BD FACSCanto II flow cytometer (BD Bioscience).
Instrument	BD FACSCanto II flow cytometer was used for data acquisition
Software	BD FACSDiva software was used for data collection and FlowJo V10 was used for data analysis
Cell population abundance	For flow cytometry analysis, peripheral blood lymphocyte was isolated from each mouse blood. 20,000 to 100,000 cells were captured during each session/sample.
Gating strategy	After excluding dead cells and cell clumps, individual cell types were identified based on the following markers:<br>Circulating tumor cells: CD45 negative, pan-cytokeratin positive<br>Peripheral blood lymphocytes: CD45 positive<br>Sample acquisition gates were created for CTCs based on the staining pattern of 6DT1 cells lines in culture which is provided in figure 8.

☒ Tick this box to confirm that a figure exemplifying the gating strategy is provided in the Supplementary Information.
